# Carvacrol and Thymol Attenuate Cytotoxicity Induced by Amyloid β_25-35 _via Activating Protein Kinase C and Inhibiting Oxidative Stress in PC12 Cells

**DOI:** 10.29252/ibj.24.4.243

**Published:** 2020-01-22

**Authors:** Zahra Azizi, Mona Salimi, Amir Amanzadeh, Nahid Majelssi, Nasser Naghdi

**Affiliations:** 1 Department of Physiology and Pharmacology, Pasteur Institute of Iran, Tehran, Iran;; 2 Department of Cell Bank, Pasteur Institute of Iran, Tehran, Iran

**Keywords:** Alzheimer’s disease, Carvacrol, Thymol, Reactive oxygen species, Protein kinase C

## Abstract

**Background:**

Our previous findings indicated that carvacrol and thymol alleviate cognitive impairments caused by Aβ in rodent models of AD. In this study, the neuroprotective effects of carvacrol and thymol against Aβ_25-35_-induced cytotoxicity were evaluated, and the potential mechanisms were determined.

**Methods:**

PC12 cells were pretreated with Aβ_25-35_ for 2 h, followed by incubation with carvacrol or thymol for additional 48 h. Cell viability was measured by the MTT method. A flurospectrophotometer was employed to observe the intracellular ROS production. PKC activity was analyzed using ELISA.

**Results:**

Our results indicated that carvacrol and thymol could significantly protect PC12 cells against Aβ_25-35_-induced cytotoxicity. Furthermore, Aβ_25-35 _could induce intracellular ROS production, while carvacrol and thymol could reverse this effect. Moreover, our findings showed that carvacrol and thymol elevate PKC activity similar to Bryostatin-1, as a PKC activator.

**Conclusion:**

This study provided the evidence regarding the protective effects of carvacrol and thymol against Aβ_25–35_-induced cytotoxicity in PC12 cells. The results suggested that the neuroprotective effects of these compounds against Aβ_25-35_ might be through attenuating oxidative damage and increasing the activity of PKC as a memory-related protein. Thus, carvacrol and thymol were found to have therapeutic potential in preventing or modulating AD.

## INTRODUCTION

Alzheimer’s disease is a neurodegenerative disorder characterized by progressive deterioration of brain structure and function^[^^1^^]^. As life expectancy is increasing in parallel with the economic development, the risk of AD along with its cost and social burden will be felt even more in the future^[^^2^^]^.

The excessive accumulation of senile plaque in the brain is one of the neuropathological hallmarks of AD^[^^3^^]^. The Aβ peptide in the plaque cores is a 39-43 amino acid sequence generated from a larger transmembrane protein, the APP^[^^4^^,^^5^^]^. APP can be processed by two alternative pathways: amyloidogenic and non-amyloidogenic. In the amyloidogenic pathway, β- and γ-secretase cooperatively convert APP into Aβ to form the deposits. In the non-amyloidogenic pathway, α-secretase reroutes the substrate APP toward the production of soluble APPα, which is non-toxic and prevents the formation of amyloid plaques^[^^5^^]^. PKC has been shown to activate α-secretase, increase the non-toxic soluble APPα release and reduce the secretion of the toxic Aβ peptide^[^^6^^]^. In this sense, PKC pharmacology would be an interesting area of research for development of cognition-enhancing agents and therapeutics against memory loss^[^^7^^]^. On the other hand, oxidative stress has been revealed to contribute to the progression of AD. Some studies have shown that Aβ plays a crucial role in the accumulation of ROS^[^^8^^,^^9^^]^. High ROS levels cause oxidative stress that may result in cell death, ultimately leading to neurodegenerative diseases including AD^[^^9^^,^^10^^]^. Therefore, therapeutic effort for attenuating the oxidative stress could be beneficial in AD treatment^[^^11^^]^.

Despite of extensive research on AD for more than a century, few effective treatment options have been developed^[^^12^^]^. Current available drugs used for the treatment of AD transiently alleviate some of the symptoms, but do not modify the disease mechanism or cure it. Therefore, there is an urgent need for the development of new drugs^[^^13^^,^^14^^]^. Nowadays, several medicinal plants and their constituents have been suggested as possible treatments for AD based on their effects on oxidant formation^[^^15^^]^ and processing of APP^[^^16^^]^. Among the chemical compounds derived from plants, monoterpenes^[^^2^^]^ along with phenolic compounds^[^^17^^,^^18^^]^ exhibit diverse pharmacological activities, representing them as potential modulators of AD. In this regard, carvacrol (2-methyl-5-(1-methylethyl) phenol)] and thymol (2-isopropyl-5-methylphenol) as two phenolic monoterpenes are abundantly found in aromatic plants, including *Thymus vulgaris* and *Zataria multiflora*^[^^19^^]^. These cyclic monoterpenes exert protective activities in a variety of pathological states including oxidative stress^[^^20^^,^^21^^]^, which might be beneficial in reducing AD symptoms.

 Previously, we investigated the effects of carvacrol and thymol in two animal models of AD and observed their effectiveness in alleviating cognitive impairments caused by Aβ^[^^22^^]^. In this study, carvacrol and thymol were used in an *in vitro* model of AD, Aβ-treated PC12 cells, and their antioxidant activity as well as their effects on PKC activity, as a memory-related enzyme, were studied. 

## MATERIALS AND METHODS

**Chemicals and agents preparation**

Aβ_25-35 _peptide, MTT, DCF-DA, DMSO, thymol and carvacrol were all provided by Sigma-Aldrich Chemical Company (St. Louis, Missouri, USA). RPMI 1640 medium, fetal bovine serum, and horse serum were obtained from Gibco BRL (Grand Island, NY, USA). PKC kinase activity kit and Bryostatin-1 were purchased from Enzo Life Sciences Company (Farmingdale, NY, USA) and Tocris Bioscience (Bristol, UK), respectively. The PC12 cell line (rat pheochromocytoma cells) was provided by the National Cell Bank, Pasteur Institute of Iran (Tehran). Aβ_25-35 _peptide was dissolved in RPMI at a concentration of 1 mM and incubated at 37 °C for 4 days to obtain the aggregated form. The solution was stored at -20 °C. The stock solution was diluted to desired concentrations immediately before use. Bryostatin-1 was dissolved in 1% DMSO at the concentration of 10 nM and stored tightly sealed at -20 °C. Carvacrol and thymol were freshly dissolved in 1% DMSO as a stock solution and then diluted with RPMI to desired various concentrations by the serial dilution of stock solution before the experiment.

**Cell culture and treatment**

PC12 cells were cultured in flasks with RPMI 1640 medium supplemented with 15% fetal bovine serum and 1% antibiotics (penicillin-streptomycin-nystatin) and incubated in a 5% CO_2 _atmosphere at 37 °C. Next, 75% of culture medium was replaced with a fresh medium every 48 h. PC12 cells were passaged when the culture was 80% confluent. The cells were subcultured once a week in the ratio of 1:4. Cell culture solutions were prepared under sterile conditions. The cells in exponential growth were seeded on 96-well plates at a density of 2 × 10^4^ cells per well, in 100 μl of growth medium and allowed to attach for 24 h. The cells were then exposed to the desired concentrations of Aβ, carvacrol, thymol, or Bryostatin-1 according to each experiment. 

**Cell viability assay**

The MTT assay was used for three different experiments. First, to achieve the appropriate concentration of Aβ, the cells were treated with different concentrations of Aβ (30, 50, and 100 μM) for 48 h. Second, to detect the safety of carvacrol and thymol, cells were exposed to different concentrations of carvacrol and thymol ranging from 1 μM to 1000 μM for 48 h. The third stage was an assay in which the cells were treated with Aβ_25-35_ (50 μM), followed by indicated concentrations of carvacrol (10, 20, and 50 μM), thymol (10, 20, and 50 μM), or Bryostatin-1 (10 nM). The control group was treated with DMSO 1% (v/v). After each of these three experiments and at the end of incubation, cell viability was evaluated by MTT assay^[^^23^^]^. In Brief, 10 µl of MTT solution, diluted in sterile PBS (5 mg/ml), was added to the culture plates. The plates were incubated at 37 °C for 4 h. Then the media was carefully removed, and 100 μl of DMSO as lysis buffer was added to each well. After 60 min, the amount of MTT formazan product was determined by measuring absorbance using a 96-well ELISA microplate reader at a wavelength of 545 nm and a reference wavelength of 630 nm. The results were expressed as the percentage of MTT reduction, assuming that the absorbance of control was 100%. All experiments were performed three independent times, each in triplicate.

**Measurement of ROS generation**

The intracellular ROS formation levels were monitored using the fluorescent probe DCF-DA, which readily diffuses through the cell membrane^[^^24^^]^. Within the cells, the nonfluorescent dye reacts with the intracellular ROS and is converted into DCF, which is a green fluorescent dye. PC12 cells were treated with 50 μM of Aβ_25-35_, followed by the various concentrations of carvacrol (10, 20, and 50 μM), thymol (10, 20, 50 μM), or 10 nM of Bryostatin-1 for 48 h. At the end of the treatment, the plates were washed twice with PBS. Then the cells were incubated in the presence of DCF-DA at a final concentration of 10 μM at 37 °C for 50 min. After incubation, DCF intensity was determined with a fluoremeter (BioTeK, Swindon, UK). The excitation and emission wavelengths were 485 nm and 528 nm, respectively. The results were expressed in relation to ROS-associated fluorescence intensity of control cells, which was set to 100%. The control group was treated with DMSO 1% (v/v). All experiments were repeated three independent times, each in triplicate.

**Sample preparation of cell lysates**

PC12 cells with different treatments were cultured in a dish (100 mm) to reach 80-90% confluence. After the removal of the culture medium, cells were washed twice with cold PBS and lysed with 300 μl/dish of cool RIPA buffer solution containing 5 M of sodium chloride, 1% Triton X-100, 0.5% sodiumdeoxycholate, 1M of Tris-HCl (pH 7.4), 0.1% SDS, supplemented with protease inhibitor. After 15-min incubation period, the plates were rotated by hand to cover cells with a film of lysis buffer. The cells were then immediately dislodged with a cell scraper, and the cell lysate were collected in a pre-chilled 1.5-ml microcentrifuge tube. In the next step, the microtubes with the cell suspensions were taped vigorously five times and leaved on ice for 15 min. Afterwards, the samples were centrifuged at 16,000 ×g at 4 °C for 15 min to separate the supernatant. At the end, the clear supernatant fractions were transferred to new pre-chilled 1.5-ml microtubes and stored at -70 °C. The protein concentration of each sample was determined using the Bradford method, and equal amounts of proteins were analyzed for PKC activity assay.

**Kinase activity assay**

The non-radioactive PKC kinase activity assay kit (ENZO, ADI-EKS-420A) was based on a solid-phase ELISA that utilizes a polyclonal antibody recognizing the phosphorylated form of the substrate. The assay was designed for the analysis of PKC activity in the solution phase. In brief, the wells of the PKC substrate microtiter plate were filled with 50 μl of kinase assay dilution buffer at room temperature for 10 minutes, and then the liquid was carefully aspirated from the wells. The samples were added to the appropriate wells of the PKC substrate microtiter plate, and the reaction was initiated by adding 10 μl of diluted ATP to each well. After 90 minutes, the reaction was stopped by discarding the contents of each well. Then 40 μl of phosphospecific substrate antibody was added to each well, and the plate was incubated at room temperature for 60 minutes. After that, the wells were washed four times with 100 μl of 1 wash buffer, and 40 μl of diluted anti-rabbit IgG, *horseradish peroxidase* conjugate, was added to each well. After 30 minutes, the wells were washed four times with 100 μl of 1 wash buffer. Then 60 μL of 3,3',5,5'-tetramethyl-benzidine substrate was added to each well, and the plate was incubated at room temperature for 30 minutes. Finally, 20 μl of acid stop solution was added to each well. The absorbance was measured at 450 nm using a Bio-Rad microtiter plate reader (USA).

**Statistical analysis**

All data were presented as means ± SEM. Statistical analyses were carried out using one-way ANOVA, followed by Tukey's honestly significant difference. *p *< 0.05 was considered to be statistically significant compared with Aβ_25-35 _treatment or control group.

## RESULTS

**Determination of Aβ**_25-35_ **cytotoxicity**

**Fig. 1:**
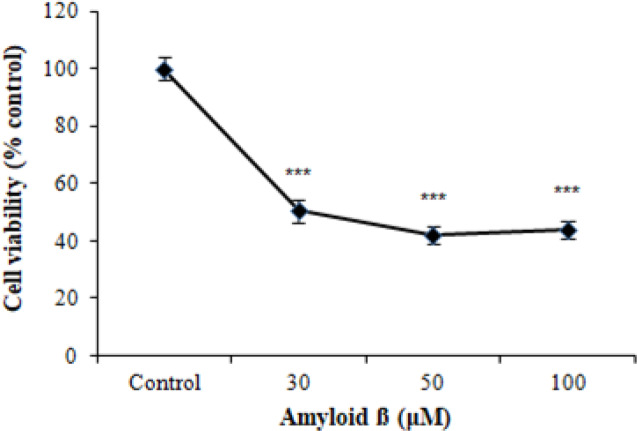
Aβ_25-35_-induced cell death in PC12 cells. The cells were incubated with different concentrations of Aβ_25-35_ (30, 50, and 100 µM) for 48 h, and the cell viability was measured by MTT assay. The data were presented as the mean ± SEM, n = 9. ^***^p < 0.001 vs. control

First, to assess the Aβ cytotoxicity, PC12 cells were treated with different concentrations (30, 50, and 100 μM) of Aβ_25-35_. As shown in Figure 1, cell viability measured by MTT assay significantly reduced after Aβ_25-35 _treatment at different concentrations of Aβ_25-35_ for 48 h. Based on our results, 50 μM of Aβ_25-35 _showed the maximum cytotoxicity, and this concentration was selected for our further experiments.

**Safety of carvacrol and thymol**

**Fig. 2:**
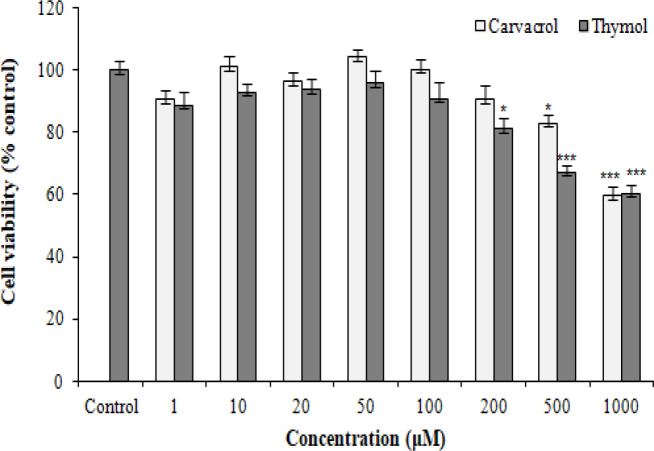
Concentration-dependent effects of carvacrol and thymol on the cell viability of PC12 cells. The cells were incubated with different concentrations of carvacrol or thymol (1-1000 µM) for 48 h, and the cell viability was measured by MTT assay. The viability of the control group (DMSO 1%) was set to 100%. The data were presented as the mean ± SEM, n = 9. ^*^p < 0.05 and ^***^p < 0.001 vs. control

The safe doses of carvacrol and thymol were first determined by measuring the cell viability assay. Carvacrol and thymol at concentrations ranging between 1 and 1000 μM were added separately to the wells containing PC12 cells for 48 h. The results showed statistically significance difference between the control group (1% DMSO), and the groups were treated with carvacrol at concentrations up to 200 μM; however, treatment with high doses of carvacrol (500 and 1000 μM) resulted in PC12 cytotoxicity. Furthermore, exposure to concentrations up to 100 μM of thymol did not decrease the cell viability compared with the control group (Fig. 2). Our findings indicated that 1-200 μM concentrations of carvacrol along with 1-100 μM concentrations of thymol revealed no cytotoxicity against PC12 cells and those used for our further experiments.

**Carvacrol and thymol protectection against Aβ-induced cytotoxicity**

**Fig. 3:**
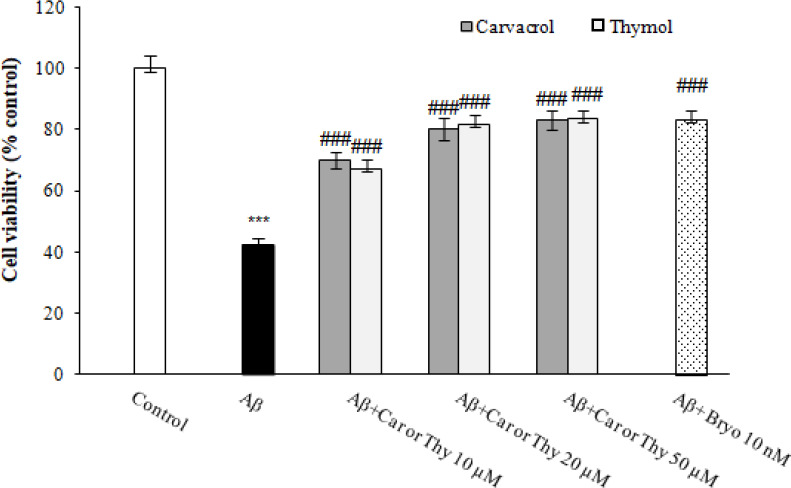
The protective effects of carvacrol (Car) and thymol (Thy) on Aβ-induced cytotoxicity in PC12 cells. The cells were incubated with different concentrations (10, 20, and 50 μM) of carvacrol and thymol and also 10 nM of Bryostatin (Bryo)-1 in the presence of 50 μM of Aβ_25-35 _for 48 h. The viability of the control group (DMSO 1%) was set to 100%. The data were presented as the mean ± SEM. ^***^p < 0.001 vs. control, ^###^*p* < 0.001 vs. Aβ

In order to explore the protective effects of carvacrol and thymol against Aβ_25-35_-induced cytotoxicity, PC12 cells were incubated with 50 μM of Aβ_25-35_, and 30 minutes later, carvacrol or thymol at concentrations of 10, 20, and 50 μM were added to the cells for 48 h. The cytotoxicity induced by Aβ_25-35 _was reversed following treatment with carvacrol at concentrations of 10, 20, and 50 μM by 70%, 80%, and 85%, respectively (Fig. 3). Similarly, thymol could reverse Aβ toxicity at concentrations of 10, 20, and 50 μM on PC12 cells. Our findings imply that treatment with carvacrol or thymol at concentrations of 10, 20 and 50 μM could reverse the cytotoxicity induced by Aβ_25-35_ on PC12 cells. This effect was similar to Bryostatin-1 that showed significant protection against Aβ_25-35_-induced cytotoxicity (Fig. 3).

**Carvacrol and thymol prevention against Aβ-induced ROS production**

**Fig. 4:**
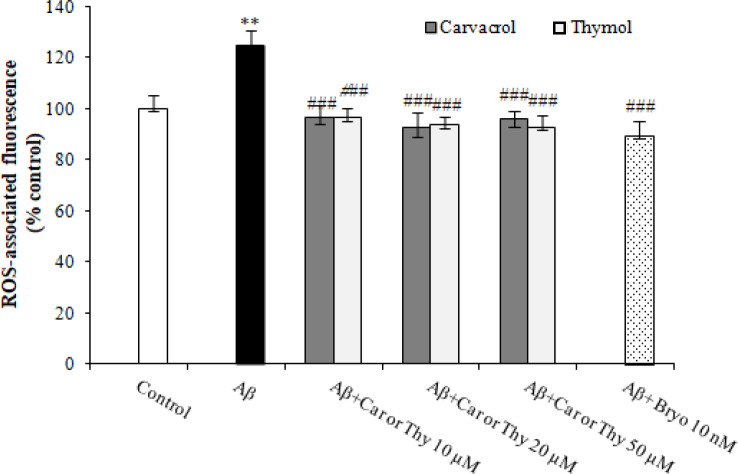
Effects of carvacrol (Car) and thymol (Thy) on the intracellular accumulation of ROS produced by Aβ_25-35 _in PC12 cells. The cells were incubated with different concentrations (10, 20, and 50 μM) of carvacrol and thymol or 10 nM of Bryostatin (Bryo)-1 in the presence of 50 μM of Aβ_25-35 _for 48 h. Intracellular ROS was measured based on the peroxide-sensitive DCF. ROS-associated fluorescence of the control group (DMSO 1%) was set to 100%. The data were presented as the mean ± SEM. ^**^p < 0.01 vs. control, ^###^*p* < 0.001 vs. Aβ group

Intracellular accumulation of ROS was determined by DCF-DA assay as described in the material and methods. A substantial increase in the level of ROS was observed (Fig. 4) in PC12 cells exposed to 50 μM of Aβ_25-35_ for 48 h compared to the control group (DMSO 1%). Also, 10 nM of Bryostatin-1 significantly reduced intracellular ROS levels compared to Aβ group. Carvacrol and thymol at concentrations of 10, 20, and 50 μM showed a similar effect as Bryostatin (Fig. 4). Altogether, the results demonstrated that carvacrol and thymol had similar behavior toward Bryostatin-1 in the reduction of ROS level. 

**Induction of PKC activity by carvacrol and thymol in PC12 cells-treated with Aβ**

**Fig. 5:**
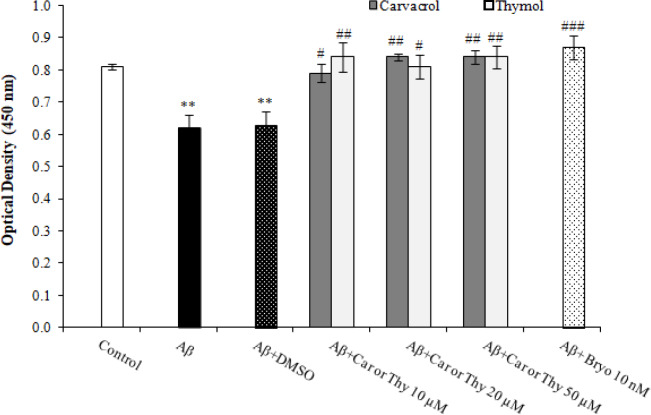
Carvacrol (Car) and thymol (Thy) effect on PKC pathway inhibited by Aβ_25-35_. The cells were incubated with 10, 20, and 50 μM of carvacrol and thymol or 10 nM of Bryostatin (Bryo)-1 following treatment of 50 μM of Aβ_25-35 _for 48 h. The phosphorylation of PKC detected in cell lysates of PC12 using an anti-phospho-PKC (pan) antibody. The data were presented as the mean ± SEM. ^**^p < 0.05 vs. control, ^#^*p* < 0.05 vs. Aβ, ^##^*p* < 0.01 vs. Aβ, ^###^*p* < 0.001 vs. Aβ

Enzyme activity was determined using an assay kit obtained from Enzo Life Science (USA). We found that exposure of PC12 cells to 50 μM of Aβ_25-35_ for 48 h considerably inhibited PKC activity compared to the control group. As expected, Bryostatin-1 significantly inversed this effect. Treatment with carvacrol or thymol at concentrations of 10, 20, and 50 μM could also significantly induce the PKC activity (Fig. 5). 

## DISCUSSION

In our previous work, we demonstrated the potential neuroprotective effects of carvacrol and thymol in AD animal models^[^^22^^]^. Our present results indicated that, applying carvacrol and thymol attenuated cell death induced by Aβ_25-35_, and prevented Aβ_25-35_-induced oxidative stress in PC12 cells. In addition, Aβ_25-35_ inhibited PKC activation, whereas carvacrol and thymol could reverse this effect similar to Bryostatin-1, as a potent PKC activator.

Aβ deposition and subsequent formation of senile plaques in the brain have well been recognized as key events during neuropathogenesis of AD. Therefore, studies on the discovery of drugs inhibiting Aβ formation and toxicity are of greatsignificance for AD treatment^[^^25^^]^. Studies have suggested that the neurobiological effects of phytochemicals derived from medicinal plants may contribute to clinical benefits in AD^[^^14^^,^^26^^]^. Carvacrol and thymol, natural monoterpenoid phenols, are obtained from many plants belonging to Lamiaceae family and possess diverse pharmacologic properties including antibacterial^[^^20^^]^, cardioprotective^[^^21^^]^, and anticancer^[^^27^^]^ effects. Neuro-protective effects of these compounds have been reported in a variety of animal models, including AD^[^^22^^,^^28^^,^^29^^]^. Herein, the effects of carvacrol and thymol on Aβ-induced cytotoxicity were evaluated in PC12 cells. The rats adrenal pheochromocytoma cells line, namely PC12, appears to be an *in vitro* cell model for neurobiology and neurochemistry studies^[^^30^^]^. PC12 cells were found to respond specifically to the 25-35 fragment of Aβ as a neurodegenerative factor in AD, stimulating the production of cellular ROS and Aβ-mediated cell death^[^^31^^,^^32^^]^. The results of MTT assay revealed that treatment with carvacrol or thymol significantly increased the cell viability compared to that of the Aβ_25-35 _group.

Intracellular oxidative stress resulting from imbalance in ROS production and cellular antioxidant defense mechanisms mediates the damage to proteins and nucleic acids, which has direct and deleterious consequences in AD^[^^33^^]^. Evidence has confirmed that the increased amounts of Aβ peptide elevate the production of ROS^[^^34^^,^^35^^]^; therefore, antioxidants may be merged as therapeutic strategies to attenuate Aβ-induced neurotoxicity and improve neurological outcomes in AD^[^^11^^]^. In this study, when the PC12 cells were incubated with Aβ_25–35_, the contents of ROS significantly increased. Meanwhile, co-treatment with carvacrol or thymol attenuated the Aβ_25–35_-induced increase of ROS levels. Therefore, the ability of these compounds to protect PC12 cells could be partially attributed to their antioxidant property. Our results are in accordance with other studies denoting that carvacrol and thymol possess strong anti-oxidative activity^[^^20^^,^^21^^]^. Many investigations have reported that antioxidant agents may slow the progression of AD^[^^10^^]^. For instance, Asadbegi *et al.*^[^^36^^]^ have disclosed that thymol, a natural antioxidant, can be therapeutic against high risk factors for AD. In the present study, administrating carvacrol and thymol could effectively ameliorate the induction of oxidative stress in PC12 cells. As oxidative stress is a process always associated with AD, carvacrol and thymol could be considered as potential candidates for AD treatment. 

PKC as a phospholipid-dependent protein kinase is a crucial player in various cellular functions either in neuronal and non-neuronal cells^[^^37^^]^. It is well established that signaling deficits of PKC pathway have an important role in the pathophysiology of neurodegenerative disorders including AD^[^^38^^,^^39^^]^. On the other hand, PKC activators are regarded as a potential candidate for the treatment of AD because of APP processing activation through α-secretase^[^^6^^,^^40^^]^. In this regard, it has been shown that Bryostatin-1 reduces amyloid plaques in the brain of transgenic AD mice, improves behavioral outcomes and consequently decreases the death rate^[^^7^^]^. Consistent with these data, our findings obtained from PKC activity assay revealed that carvacrol and thymol reversed Aβ-triggered PKC inhibition, representing a new mechanism of action for carvacrol and thymol in reducing Aβ-induced neurotoxicity. Interestingly, Bryostatin-1 acted in the similar way, suggesting the possible modulating role for carvacrol and thymol in PKC activity.

The results of the current study revealed that the protective effects of carvacrol and thymol against Aβ-induced cytotoxicity are not only attributable to their antioxidant properties but also they may occur through other mechanisms such as PKC stimulation. In accordance with our results, Chen *et al.*^[^^41^^]^ have displayed that the cardioprotective effects of carvacrol may be attributed to its antioxidant property through the activation of the MAPK/ERK signaling pathway, as the common downstream effectors of PKC^[^^40^^]^. Therefore, considering the possible neuroprotective role of PKC in AD, it is reasonable to suggest administration of carvacrol and thymol as potential disease-modifying agents.

Given the involvement of multiple factors in the pathogenesis of AD as well as the aberrant processing of APP, AD treatment requires multiple drug therapies to address the varied pathological aspects. New potential approaches have been developed to target multiple sites in the brain with single molecular entities for treatment of AD^[^^42^^,^^43^^]^. The results of the present study showed that carvacrol and thymol may present multi-functional properties, including antioxidant activity and PKC activity modulation. Our results are consistent with a number of previous studies, introduced numerous bio-molecules belonging to terpenoid^[^^44^^]^ or phenolic structures^[^^45^^,^^46^^]^ as novel therapeutic candidates with the properties such as radical scavenging and PKC activation.

 Our results provide evidence for the neuroprotective effect of carvacrol and thymol against cytotoxicity induced by Aβ_25-35_. The underlying mechanism might be through decreasing ROS formation as well as stimulating PKC activity. Taken together, these two compounds may have therapeutic potential in preventing or modulating AD. However, further studies are required to clarify all possible neuroprotective mechanisms of carvacrol and thymol in AD models. 
